# Defensins: The Case for Their Use against Mycobacterial Infections

**DOI:** 10.1155/2016/7515687

**Published:** 2016-09-20

**Authors:** Haodi Dong, Yue Lv, Deming Zhao, Paul Barrow, Xiangmei Zhou

**Affiliations:** ^1^National Animal Transmissible Spongiform Encephalopathy Laboratory, Key Laboratory of Animal Epidemiology and Zoonosis of Ministry of Agriculture, College of Veterinary Medicine and State Key Lab of Agrobiotechnology, China Agricultural University, Beijing, China; ^2^School of Veterinary Medicine, University of Nottingham, Sutton Bonington, Loughborough, Leicestershire Le12SRD, UK

## Abstract

Human tuberculosis remains a huge global public health problem with an estimated 1/3rd of the population being infected. Defensins are antibacterial cationic peptides produced by a number of cell types, most notably neutrophil granulocytes and epithelial cells. All three defensin types (*α*-, *β*-, and *θ*-defensins) have antibacterial activities, mainly through bacterial membrane permeabilization. Defensins are effective against Gram-negative and Gram-positive bacteria including mycobacteria and are active both intra- and extracellularly. Mycobacterial resistance has never been demonstrated although the* mprF* gene encoding resistance in* Staphylococcus aureus* is present in the* Mycobacterium tuberculosis* genome. In addition to their antibacterial effect, defensins are chemoattractants for macrophages and neutrophils. There are many cases for their use for therapy or prophylaxis in tuberculosis as well. In conclusion, we propose that there is considerable scope and potential for exploring their use as therapeutic/prophylactic agents and more comprehensive survey of defensins from different species and their bioactivity is timely.

## 1. Introduction

Tuberculosis remains the most important infectious disease globally, and* Mycobacterium tuberculosis* is thought to be present in one-third of the world's population with 8–10 million new cases of active tuberculosis occurring annually worldwide. In 2013, an estimated 9.0 million people developed tuberculosis, and 1.5 million died from the disease [1]. Almost all cases of tuberculosis are caused by* M. tuberculosis*, with* M. bovis* contributing less than 1.4% of all pulmonary cases outside of Africa and ~2.8% of cases in Africa with a crude incidence of 7 cases per 100,000 population [2]. The incidence of disease in some countries is also exacerbated by HIV infections [1]. Because of the difficulties of chemotherapy, the incidence of multidrug-resistant strains of* M. tuberculosis* has increased in many areas during recent decades [1]. This deterioration in the global situation highlights the need for new therapeutic agents against mycobacterial diseases. Recent research has shown that defensins have bactericidal activity against* M. tuberculosis* and indicate their potential to play a more significant role in tuberculosis control than what was previously considered [3].

Protective immunity against mycobacterial infections requires the generation of an effective cell-mediated immunity [4]. However, an efficient innate immune response may also be important in natural resistance against mycobacterial infection in addition to maintaining longer-term control of bacillary growth during latent infection. Alveolar macrophages and lung epithelial cells are the main cells to first encounter* M. tuberculosis* during primary infection. Studies of human airway epithelia* in vivo* or* in vitro* show that they can generate antimicrobial activity through the production of antimicrobial peptides [5, 6]. In addition, defensins account for a high proportion of the antibacterial activity associated with neutrophil granules [7, 8]. Defensins act as a bridge linking innate and acquired immunity largely through their chemotactic properties. They belong to a family of small (3–5 kDa) cationic cytotoxic and oxygen-independent peptides that are active against a wide spectrum of microorganisms including bacteria, viruses, and fungi [9]. This review presents a brief summary of their role in immunity with specific reference to human and animal tuberculosis and explores their potential as a novel approach to therapy or prophylaxis.

## 2. Defensins in Man and Animals

There are three major classes of defensins expressed by different cells within the vertebrate world; *α*-, *β*-, and *θ*-defensins are differentiated by their structure and antimicrobial activity.

*α*-defensins, which have been identified in humans, monkeys, and several rodent species, are particularly abundant in polymorphonuclear neutrophils (PMNs), certain macrophage populations, and Paneth cells of the small intestine [8, 10, 11]. Human neutrophil *α*-defensins (HNPs) 1–4 constitute ~30% of proteins in the azurophilic granules of PMNs [8]. However, against Gram-positive bacteria, HNP-1, HNP-2, and HNP-3 account for most of the total defensin content and have greater antimicrobial activity than HNP-4 [12]. In contrast, the potency of HNP-4 against Gram-negative bacteria is greater than those of HNP-1 and HNP-2 [13]. *α*-Defensins 5 and 6 are found in Paneth cells, the epithelial granulocytes of the small intestine [14, 15]. *α*-Defensins are also found in alveolar macrophages from rabbits but not from humans [16]. *α*-Defensins show activity* in vitro* against Gram-negative and Gram-positive bacteria and fungi [12] that can be modulated by environmental conditions such as redox and pH [17].

In contrast to *α*-defensins, *β*-defensins are largely expressed in epithelial tissues [18, 19]. The first *β*-defensin was identified in bovine tongue tissue, and *β*-defensins subfamilies have now been reported in primates (humans and apes), bovines, and rodents such as rats and mice [6, 20]. Furthermore, pigs and other farm animals including birds express only *β*-defensins [20–22]. As the most comprehensively studied, *β*-defensins possess the widest taxonomic distribution, including invertebrates and plants, indicating an ancient point of origin [23]. Human *β*-defensin 1 (HBD-1) is expressed constitutively by a number of body systems including the urogenital tract and respiratory tract [24, 25]. HBD-2 was discovered in extracts of lesional scales from patients suffering from psoriasis [26] and is expressed by inflamed skin, lung, oral mucosa, and ocular surfaces [27]. Its expression by epithelial cells can be induced by TNF-*α* [28] and interleukin- (IL-) 1*β* but also by bacteria [18]. HBD-3 is expressed mainly by the skin and tonsils [29], and HBD-4 is expressed by many tissues but is particularly highly expressed in the gastric antrum and testes [30]. The expression of HBD-1 is constitutive, whereas HBD-2–4 are inducible [31] and are thought to play a crucial role against bacterial infection as part of the epithelial barrier [32]. Proinflammatory cytokines, bacteria, and fungi have all been found to increase the expression of these defensins in cultured keratinocytes [20].

*θ*-Defensins are the only defensin subfamily with a circular structure, which likely originated through mutation of a preexisting *α*-defensin gene in Old World monkeys [33]. They were first found in the monocytes and neutrophils of the rhesus monkey (*Macaca mulatta*) and are the least numerous subfamily of mammalian defensins [34], with only three *θ*-defensins recognized (rhesus theta defensin (RTD) 1–3) from studies within leukocytes [34]. Circular defensin isoforms also exist with five identified in peripheral blood leukocytes and four in the bone marrow in the olive baboon (*Papio anubis*). Less is known about the distribution and diversity of *θ*-defensins because only 11 different *θ*-defensins have been isolated from three species of primates since their discovery in 1999 [35]. The antimicrobial activity of *θ*-defensins is thought to be their natural function [35], and they bind to and neutralize bacterial toxins [36, 37]. For example, the human *θ*-defensin retrocyclin-1 and its analogues are active against* C. albicans* and* L. monocytogenes* and bind to anthrax lethal toxin [37].

To date, more than 17 human defensins have been reported [10]. However, many more *β*-defensins have been reported by* in silico* analysis [38], and a genome-wide computational search has identified more than 30 human uncharacterized *β*-defensin genes with full biological significances currently undetermined [39].

## 3. Mechanism of Action; the Role of Defensins in Immunity

### 3.1. Chemotaxis

The role of defensin chemotactic activity in initiating and regulating the immune response is now well known [30–32]. The human *α*-defensin family chemoattracts macrophages [8]. Amongst *β*-defensins, they possess chemotactic activities for immature memory T-cells and dendritic cells through the chemokine receptor CCR6 [40]. HBD-2 and HBD-3 can combine with both CpG and host DNA to form aggregates that resemble DNA nets, which may enhance the intracellular uptake of CpG and self DNA and activate plasmacytoid dendritic cells (pDCs) to promote DNA-induced IFN-*α* production in a Toll-like receptor 9- (TLR9-) dependent manner [41]. Subcutaneous injections of these complexes showed enhanced infiltration of inflammatory cells at the injection site, indicating a potential pathophysiological role for defensin/DNA complexes in contributing to inflammation [41]. A recent study showed that murine *β*-defensin 2 (mBD-2) immunostaining in tuberculous mice was essentially localized to cells with dendritic morphology located near the mediastinal lymph nodes and showed a high level of gene expression [42]. This suggests that *β*-defensins may play an important role in the initiation of a Th1 response as a link between the innate and adaptive immune responses [42]. HNPs can also promote B- and T-cell interactions by modulating the Th1- and Th2-type cytokines [43, 44]. *θ*-Defensins have not been found to possess chemotactic activity.

The expression of HBD-2 by human macrophages can be triggered by* M. tuberculosis* [45]. The expression and production of defensins are activated by a number of routes including direct recognition of pathogen-associated molecular patterns (PAMPs), such as LPS, by TLR [46]. This initiates MAPK- or NF-*κ*B-dependent cascades that culminate in a proinflammatory response involving the secretion of cytokines, chemokines, and defensins [19], which themselves also have the capacity to induce defensin secretion. Their expression is also mediated by receptors other than TLRs, including NOD2 [47]. Thus, infection of cells with live* Mycobacteria* leads to the induction of TNF*α* and HBD-2 [48].

Defensins are also thought to contribute to the inflammatory processes by inducing histamine release by mast cells and increasing hyperresponsiveness of the airways to histamine [49]. Besides their antimicrobial and potential proinflammatory activities, defensins also display anti-inflammatory roles by binding to C1q to inhibit activation of the classical pathway of complement activation [49] and have been demonstrated to inhibit fibrinolysis [50]. Some defensins are also able to limit the inhibitory action of glucocorticoids on the suppressor functions of T-lymphocytes, which was abolished after adrenalectomy [51].

### 3.2. Antibacterial Activity

The main mode of antibacterial action is the direct lysis of microorganisms through permeabilization of cell membranes [8] either whilst the bacteria are extracellular or after phagocytosis [52]. It is believed that electrostatic binding between the arginine groups of cationic defensins and membranes rich in anionic phospholipids induces the formation of voltage-regulated channels, which causes the leakage of intracellular metabolites [8]. Some defensins can also bind avidly to membrane glycoproteins [53], which may be in part responsible for their antiviral activity. This is supported by the fact that defensins do not have antiviral activity against nonenveloped viruses [8].

In addition, defensins can bind to polyanionic molecules such as DNA via electrostatic interaction after entering the bacterial cell [54]. Since HNPs also target genomic DNA, inducing single-strand DNA breaks [55], it has been hypothesized that adenosine 5′-diphosphate ribosylation might play a regulatory role in the biological properties of arginine-rich HNPs [56]. Defensins are highly antibacterial even in micromolar concentrations for both Gram-negative and Gram-positive bacteria, including mycobacteria. Unfortunately, the relative antibacterial activity between *α*- and *β*-defensins is still unknown.

### 3.3. Bacterial Resistance to Defensins and Cytotoxicity

Pathogens that colonize sites where defensins may be present in high concentrations have developed mechanisms to resist their antibacterial activity. A number of genes responsible for defensin resistance have been identified in different pathogens. A staphylococcal gene,* mprF*, confers resistance to several antimicrobial peptides including defensins and related genes and has been found in the genomes of several other pathogens such as* M. tuberculosis*,* Pseudomonas aeruginosa*, and* Enterococcus faecalis* [57]. MprF reduces the negative charge of the membrane surface and leads to decreased binding of the cationic defensins by modifying phosphatidylglycerol with L-lysine in the membrane lipids [57]. The* phoP* gene in* Salmonella enterica* serovar Typhimurium is also thought to contribute to defensin resistance because a* phoP* mutant shows significantly greater sensitivity to defensins [58].

Potential clinical application of defensins must thus be reviewed in the light of possible development or acquisition of resistance. However, their nonspecific mode of action suggests that they should show promise in averting the development of resistance. Moreover, studies have demonstrated that resistance is less frequent than that observed for conventional antibiotics [59–61], and selections for resistance in susceptible strains of* M. tuberculosis*,* Pseudomonas aeruginosa*, and* Enterococcus faecalis* have failed [62].

In addition to their toxic effects on microorganisms, there are a number of reports indicating cytotoxicity for eukaryotic cells. High concentrations of HNPs are found in the airway secretions of patients with chronic inflammatory lung disorders. It has been reported that HNPs are cytotoxic to airway epithelial cells and can induce chemokine secretion in several cell types such as macrophages [63]. Other studies showed that HNPs are cytotoxic not only against various kinds of human and murine tumor cells but also against a wide range of normal cells, including human endothelial cells, lymphocytes, murine thymocytes, PMNs, and spleen cells in a concentration-dependent (25–100 *μ*g/mL) and time-dependent manner [62, 64]. As cytotoxic molecules, HNPs can cooperate with hydrogen peroxide, which is also secreted by activated neutrophils, to affect synergistic cytolytic activity* in vitro*. This interaction may contribute to granulocyte-induced cytotoxicity* in vivo* [59, 65].

Some studies showed that the minimum inhibitory concentration and median inhibitory concentration of HNP-1 were 2.5 *μ*g/mL and 0.8 *μ*g/mL, respectively, which are much lower than the harmful concentrations to normal cells [60]. This indicates that defensins have a relatively low level of cytotoxicity to normal cells at antimicrobicidal concentrations. Low cytotoxicity* in vitro* might be due to the presence of fetal bovine serum in the culture media, as serum proteins can protect mammalian target cells [61]. The inhibitory effect of fetal calf serum (FCS) on lysis and binding can be completely accounted for by its content of albumin [61]. Not only could FCS prevent defensin binding, but also it removed membrane-bound defensin molecules from the targets [61]. Several proteins that can bind to defensins have been identified, and some of them may work as defensin carriers for clearance from tissues and blood [32].

## 4. Antimycobacterial Activity

A high concentration of *α*-defensins has been detected in bronchoalveolar lavage (BAL) samples and pleural fluid from patients suffering from pulmonary tuberculosis, and significant levels of *β*-defensins have been detected in bronchoalveolar lavage fluid from patients with* M. avium*-*intracellulare* infection [66]. Moreover, studies of gene expression profiles by microarray of peripheral blood mononuclear cells (PBMC) from patients suffering from tuberculosis and* M. tuberculosis*-infected healthy individuals who had repeated close contact to tuberculosis patients (such as a nurse, physician, or family member) and were tuberculin skin test positive showed that the concentrations of effector molecules, *α*-defensins 1, 3, and 4, were upregulated in the diseased patients [67]. Furthermore, mice infected with 1.5 × 10^4^ CFU of* M. tuberculosis* H37Rv and treated with different doses of HNP-1 injected subcutaneously showed significantly improved clearance of bacilli from the lungs, liver, and spleen [68]. HNP-1 at 5 *µ*g/mL killed* M. avium-intracellulare in vitro* at the optimal pH for bactericidal activity (>5) [69]. The minimal inhibitory concentration (MIC) of HNP-1 against* M. tuberculosis in vitro* in one study was 2.5 *μ*g/mL [60], much lower than that reported in a second study (25 *μ*g/mL) [70]. The difference in MIC observed could be attributed to differences in experimental design.

HNP was the first defensin found to be effective against nontuberculous mycobacteria, including* M. avium*-*intracellulare* [69]. HNP-2 and HNP-3 were as effective in killing* M. avium*-*intracellulare in vitro* as HNP-1 [69]. For HNP-5, the linear peptide derived from its N-terminal fatty acylation can enhance activity against* M. tuberculosis* almost comparable to the native peptide [71].

Bovine and rabbit defensins have similar or more potent antimycobacterial activity than HNPs, especially against* M. tuberculosis* clinical isolates* in vitro* [72, 73]. In contrast, another study showed that HNP-1–3 are not necessarily more effective in killing* M. tuberculosis* even at a much higher concentration, which may be the result of differing levels of resistance in the individual strains (*M. tuberculosis* Erdman) used [74]. Defensins may play a much more significant role in immunity against mycobacteria than what was previously thought. One can therefore speculate that defensin production, especially by epithelial cells and neutrophils, is likely to be more important early in infection before the establishment of the granuloma.

Based on its antimicrobial activity and function in host immunity, neutrophil-macrophage cooperation against* M. tuberculosis* may thus be involved in clearance [75] based on the following rationale. First, HNPs clearly show antimicrobial activity against* M. tuberculosis in vitro* by increasing the permeability of the mycobacterial cell envelope [76]. Second, in addition to direct antimicrobial activity, HNPs secreted by neutrophils recruited into the early lesion are clearly able to act as chemotactic factors, attracting immune cells including macrophages, T-lymphocytes, mast cells, and immature memory T-cells [8]. Third, HNPs released by neutrophils recruited in the early lesion can also modulate cytokine production to influence the inflammatory response since TNF-*α* secreted by macrophages may stimulate neutrophil mycobactericidal activity, which might be mediated simultaneously by defensins [74].

*In vitro*, the capacity of macrophages to control* M. tuberculosis* growth is improved by transfecting human monocyte-derived macrophages with the HBD-2 gene compared with non-HBD-2-transfected cells [77]. HBD-2 [78] shows great antimicrobial activity against* M. tuberculosis* H37Rv* in vitro* [78]. Like HBD-2, HBD-1 also plays a role in immunity against* M. tuberculosis* by permeabilization of both the mycobacterial cell wall and the cell membrane [79].* M. tuberculosis* infection of endothelial cells* in vitro* also results in HBD-1 overexpression and profound cytoskeletal rearrangement [80]. One study found that infection of human limbocorneal fibroblasts with* M. tuberculosis*,* M. abscessus*, and* M. smegmatis* results in overexpression of HBD-1–3 [48]. Recent studies have emphasized the role of HBD-4, which can be triggered through the IL-1*β* and vitamin D receptor (VDR) pathways in the innate immune defense against* M. tuberculosis* survival in infected macrophages [81]. One study showed that intratracheal administration of L-isoleucine into mice infected with the antibiotic-sensitive strain H37Rv or a multidrug-resistant clinical isolate can significantly upregulate *β*-defensins 3 and 4 and decrease bacillary loads by inducing their gene expression [82], demonstrating that it may be possible to use defensins for treating infection by modulating their gene expression.

Interestingly, more highly virulent* M. bovis* strains induce lower levels of murine *β*-defensin 4 (mBD4) expression than strains of lower virulence during many time points of early infection [83], indicating the ability to suppress induction early in infection* in vivo*. In experimental tuberculosis, expression of mBD3 and mBD4 by airway epithelial cells in the early stages of infection correlated with temporary control of mycobacterial growth [84]. Similarly, high and stable production mBD4 during latent infection is associated with long-term control of mycobacterial proliferation [83]. The activity of defensins against mycobacteria coupled with their induction by infection suggests that the introduction of defensins either prophylactically or early in infection may affect the course of the disease to the benefit of the host. There thus exists the potential for the use of defensins as new prophylactic/therapeutic agents against mycobacterial infections.

## 5. Antimycobacterial Therapy

The effective* in vitro* activity of a number of defensins against mycobacteria combined with their beneficial chemotactic effects suggests that therapeutic or prophylactic administration of defensins or their induction in the body might lead to improvement in the course of infection and host health.

To date, there are relatively few reports on the effects of defensin administration against* M. tuberculosis*, but most of these show beneficial effects. Mice transfected with the *β*-defensin 2 gene showed higher survival and lower bacterial burden after challenge [85]. HNP-1 injected postinfection showed significant time- and dose-dependent clearance of bacilli from lungs, livers, and spleens in mice experimentally infected with* M. tuberculosis* H37Rv [68]. The HNP-1 administered to mice in that study was significantly less (1 and 5 *μ*g per mouse) than the concentration required (50 *μ*g/mL) for antimycobacterial activity* in vitro*. The higher* in vivo* potency of HNPs is likely due to their immune enhancing effects, such as chemotaxis of T-cells [86] and monocytes [87].

Human defensins are reported to show synergistic activity with antituberculosis drugs, which suggests that they may be a promising adjunct to antituberculosis chemotherapy [88]. A number of studies have explored the combined effect of defensins and antituberculosis drugs against intracellular mycobacteria.* In vitro* studies suggest that HBD-2 is involved in reducing* M. tuberculosis* growth, and the combination of HNP-1 with antituberculosis drugs (i.e., isoniazid and rifampicin) resulted in a significant reduction (*P* < 0.001) in mycobacterial load [76]. HBD-1 has a lower activity against* M. tuberculosis*, and its combination with isoniazid significantly reduced* M. tuberculosis* growth in comparison with the peptides or isoniazid alone by permeabilization of both the mycobacterial cell wall and the cell membrane [79]. Moreover, a protective role for *α*-defensin against mycobacterial infection has been reported in human eosinophils [89]. *α*-Defensin released by eosinophils upon stimulation with lipomannan from* M. bovis* BCG, when used with eosinophil cationic protein, showed a synergistic inhibitory effect on mycobacterial growth inhibition [89].

Since the peculiar mycobacterial cell envelope is considered to contribute to the resistance to conventional antimycobacterial drugs, the combination of HNPs and antituberculosis drugs against* M. tuberculosis* H37Rv not only results in increased permeability of both the mycobacterial cell wall and the cell membrane but also increases the access to intracellular targets for antituberculosis drugs. Therefore, antimicrobial peptides are potential adjuncts to chemotherapy together with conventional drugs against tuberculosis. Antimicrobial peptides can be more potent* in vivo* because of their immune enhancing effects by acting as a chemotactic factor and regulatory factor, interacting with immune cells like T-cells and monocytes and modulating the production of cytokines and inflammation.

Studies have shown that* M. bovis* BCG-induced HBD-2 mRNA expression in human epithelial cells can influence protection against* M. tuberculosis* challenge [90], and* M. bovis* BCG cell wall components (18–30 kDa) can stimulate human pulmonary epithelial cells to express defensins [91]. It also shows that prime-boost BCG vaccination with *β*-defensin 2 DNA vaccines can enhance the activity against* M. tuberculosis* [85]. It is known that protection conferred by BCG against tuberculosis is variable and can maintain long-term immunity [92], and defensins could be important as a component part of this protection against human tuberculosis.

Although *θ*-defensins have antimicrobial activity against diverse pathogens [35, 93], especially viruses [94], there is, as yet, no evidence that *θ*-defensins are involved in defense against mycobacterial infection.  Table 1 shows a summary of different defensins and there function in antimycobacterium.

## 6. Future Perspective

Previous studies have widely demonstrated that defensins have activities against microorganisms including* mycobacteria*. Rivas-Santiago et al. found that vitamin D and L-isoleucine can induce the production of defensins by modulating their gene expression [82, 95]. Therefore, future studies should focus on the mechanism by which defensin gene expression is modulated. On the other hand, although a large number of studies have discovered the antimycobacterial activity of defensins* in vitro*, there are fewer studies* in vivo*, and further study should include the activity of defensins against tuberculosis* in vivo*. Although defensins have been examined for their clinical treatment of infections with no success, defensins have a huge clinical potential, and more research into their application is needed.

## 7. Conclusion

Defensins are a family of antimicrobial peptides that are abundant amid an array of oxygen-independent antimicrobial proteins and peptides in neutrophil granules and secreted by epithelial cells. They are effective against a wide spectrum of microorganisms including mycobacteria.

The advantages and disadvantages of the various forms of defensin therapy/prophylaxis against mycobacterial infection of man and animals outlined above indicate that this could be an effective new approach to treatment and prevention of these chronic infections, which are becoming increasingly intractable to chemotherapy. Administration of defensins may have direct effects on the pathogens, stimulate innate and adaptive immunity, or be used synergistically with currently used or new chemotherapeutic agents. The experimental work with mycobacterial infections combined with their wide spectrum of activity suggests that bacterial infections other than those caused by mycobacteria may also be amenable to this approach.

## Figures and Tables

**Table 1:** Relevance of defensins in tuberculosis.

Defensins	Source	Importance against mycobacteria	Reference
Human neutrophil *α*-defensin 1 (HNP-1)	Polymorphonuclear neutrophils, macrophages, Paneth cells	Induced by mycobacterial infection and reduces the bacteria load. The application with antituberculosis drugs	[8, 10–12, 60, 67–70, 74, 86, 87]

Human neutrophil *α*-defensin 2 (HNP-2)	Polymorphonuclear neutrophils, macrophages, Paneth cells	Kills both tuberculosis and nontuberculous mycobacteria effectively by increasing cell wall and membrane permeability. Reduces the therapeutic dosage of drugs	[8, 10–13, 69, 74, 86, 87]

Human neutrophil *α*-defensin 3 (HNP-3)	Polymorphonuclear neutrophils, macrophages, Paneth cells	Reduces the tuberculosis and nontuberculous mycobacterial load *in vitro *and *in vivo*	[8, 10–12, 69, 74, 87]

Human neutrophil *α*-defensin 4 (HNP-4)	Polymorphonuclear neutrophils, macrophages, Paneth cells	Shows antimicrobial activity against *M. tuberculosis in vitro* by increasing the permeability of the mycobacterial cell envelope and the immune enhancing effects. Shows synergy with antituberculosis drugs	[8, 10–13]

*α*-defensin 5	Paneth cells, alveolar macrophages	Shows antimicrobial activity; the linear peptide from it can enhance activity against *M. tuberculosis*	[15–17, 71]

*α*-defensin 6	Paneth cells, alveolar macrophages	Shows antimicrobial activity	[14]

Human *β*-defensin 1 (HBD-1)	Epithelial cells	Induced by mycobacteria infection and shows antimicrobial activity on stimulation with mycobacteria. Combination with isoniazid significantly reduced *M. tuberculosis*	[24, 25, 31, 48, 79, 80]

Human *β*-defensin 2 (HBD-2)	Epithelial cells	Improves the capacity of macrophages to control *M. tuberculosis.* Greatest antimicrobial activity. Combination with Bacillus Calmette-Guerin (BCG) can enhance its activity	[18, 19, 26–28, 31, 41, 42, 45, 48, 76–78, 85]

Human *β*-defensin 3 (HBD-3)	Epithelial cells	Induced by mycobacteria infection and shows antimicrobial activity and can be stimulated by L-isoleucine when infected with tuberculosis. Related to long-term control of mycobacterial infection	[29, 31, 32, 41, 48]

Human *β*-defensin 4 (HBD-4)	Epithelial cells	Stable production during latent infection and correlated with long-term control of mycobacterial infection	[30–32, 81]

*θ*-Defensins 1–3	Monocytes, neutrophils	N/A	[33–37, 93, 94]
